# Locomotion Control of Cyborg Insects by Charge-Balanced Biphasic Electrical Stimulation

**DOI:** 10.34133/cbsystems.0134

**Published:** 2024-07-05

**Authors:** Zhong Liu, Yongxia Gu, Li Yu, Xiang Yang, Zhiyun Ma, Jieliang Zhao, Yufei Gu

**Affiliations:** ^1^ School of Computing and Artificial Intelligence, Beijing Technology and Business University, Beijing 100048, China.; ^2^ School of Mechanical Engineering, Beijing Institute of Technology, Beijing 100081, China.; ^3^ New York University Abu Dhabi, Abu Dhabi 129188, United Arab Emirates.

## Abstract

The integration of electronic stimulation devices with insects in the context of cyborg insect systems has great application potential, particularly in the fields of environmental monitoring, urban surveillance, and rescue missions. Despite considerable advantages compared to the current robot technology, including flexibility, durability, and low energy consumption, this integration faces certain challenges related to the potential risk of charge accumulation caused by prolonged and repetitive electrical stimulations. To address these challenges, this study proposes a universal system for remote signal output control using infrared signals. The proposed system integrates high-precision digital-to-analog converters capable of generating customized waveform electrical stimulation signals within defined ranges. This enhances the accuracy of locomotion control in cyborg insects while maintaining real-time control and dynamic parameter adjustment. The proposed system is verified by experiments. The experimental results show that the signals generated by the proposed system have a success rate of over 76.25% in controlling the turning locomotion of cyborg insects, which is higher than previously reported results. In addition, the charge-balanced characteristics of these signals can minimize muscle tissue damage, thus substantially enhancing control repeatability. This study provides a comprehensive solution for the remote control and monitoring of cyborg insects, whose flexibility and adaptability can meet various application and experimental requirements. The results presented in this study lay a robust foundation for further advancement of various technologies, particularly those related to cyborg insect locomotion control systems and wireless control mechanisms for cyborg insects.

## Introduction

Insects, as a widely distributed species in natural ecosystems, have evolved distinctive physiological structures over millions of years, facilitating various activities, such as walking [1,2], jumping [3], climbing, flying [4–7], and interaction with the external environment [8–10]. However, traditional robots face a range of limitations, especially in terms of a lightweight structure, adaptation to complex environments, and intricate movement execution [11]. Therefore, combining the advantages of biological organisms with the programmability of robots could address the problem of myriad constraints in the existing robotic systems [12].

Rapid advancements in electronics, micro-manufacturing, sensor technologies, and bioengineering achieved in recent years have played a pivotal role in promoting the fusion of insects and robots. These breakthroughs have facilitated a more profound investigation into the physiology and behavioral patterns of insects, culminating in the development of sophisticated, compact, and robust mechatronic devices. These devices are frequently consolidated and designed in a backpack configuration, which is securely affixed to the insect's body. The backpacks serve to stimulate targeted regions of the insect's anatomy, including the brain [13,14], cerci [15], antennae [16], elytra [17,18], ganglia [19], and muscles [20,21], thereby eliciting specific movements. This stimulation elicits specific movements and effectively integrates the insects' natural capabilities with technological enhancements [22].

At present, these cyborg insects have been extensively deployed across a variety of applications, including urban search and rescue operations [23], environmental monitoring, and inspections in hazardous areas [16,23–27]. Their unique combination of biological and artificial systems offers a novel approach to addressing challenges in these domains, showcasing the potential of cyborg insects for real-world scenarios.

Extensive efforts have been made to overcome the limitations in the power dynamics of micro-robots by integrating mechanical and electronic technologies with insects [28,29] and using electrodes and other sensors to control the movement of insects [30]. In addition, with the recent progress in materials science and bioengineering, various advanced biomaterials have been used to manufacture stimulating electrodes for cyborg insects [31–34]. These innovations provided cyborg insects with greater flexibility and enhanced practicality [35].

Nevertheless, the application of stimulation currents can lead to the accumulation of residual charges at the electrode–electrolyte interface, potentially giving rise to direct current generation. Irreversible damage to neural tissues, electrode corrosion, and the production of toxic by-products can be the result of this phenomenon [25]. Electrochemical impedance spectroscopy (EIS) has been utilized by researchers to characterize the interface impedance of electrodes [36]. Through EIS analysis, parameters of the equivalent circuit model, such as the charge transfer resistance (*R*_ct_) and double-layer capacitance (*C*_dl_), can be estimated. These parameters are crucial for understanding the mechanisms of charge transfer between the electrode and tissue.

Therefore, biphasic signals have been utilized in some studies for turning control experiments in cockroaches, achieving higher control success rates compared to monophasic signals [37,38]. However, there remains a paucity of in-depth research on the control potential of biphasic electrical signals. To address the limitations of existing solutions, this study adopts balanced charge technology and proposes the use of biphasic stimulation signals for insect locomotion control. This approach aims to mitigate the cumulative damage to the insect's sensory organs caused by electrical stimulation, thereby enhancing the stability and reliability of cyborg insect locomotion control. Specifically, this study has designed a wireless control system with multifunctional output capabilities, offering a variety of stimulation parameters and multi-channel combined stimulation modes.

In practical demonstrations, the Madagascar hissing cockroach was utilized as a living insect platform. A wireless control system, acting as a “backpack”, was attached to the cockroach’s back to carry out turning control experiments. Directional movements were induced by stimulating the cockroach’s cerci through the outputs from the backpack, thus confirming the efficacy of the proposed system. The experimental results suggest that the size and weight of the proposed system are well-suited for the insect platform, and it is observed that the biphasic electrical stimulation signals output by the control system demonstrate higher reliability when compared to monophasic signals. The valuable insights provided by the findings of this study on controlling cyborg insect motion through charge-balanced electrical stimulation are not only useful for further research but also offer key experiences for advancing the technology of cyborg insects.

## Materials and Methods

### Study animal

**Fig. 1.**
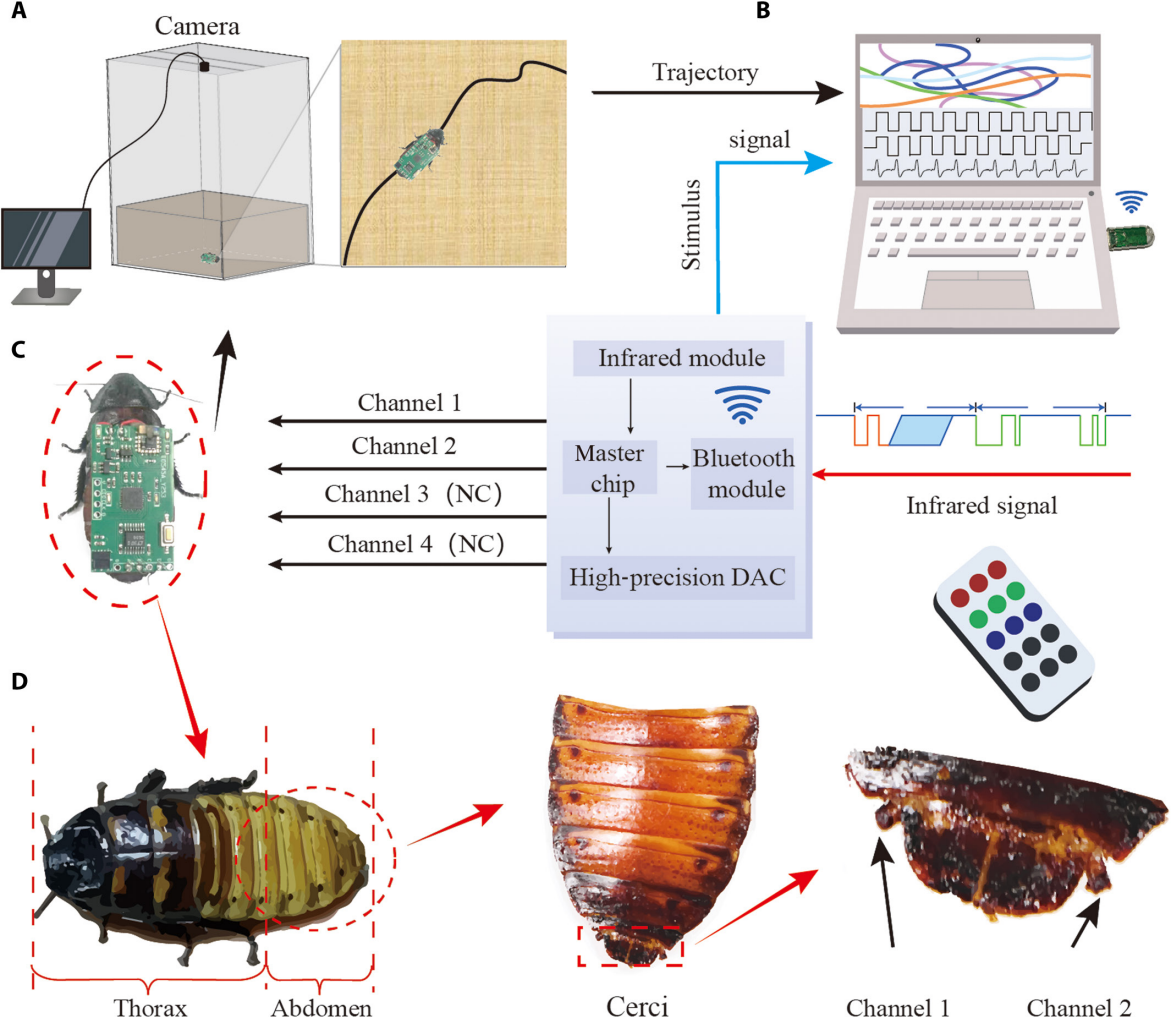
The cyborg insect is composed of the Madagascar hissing cockroach and a wireless stimulation backpack on its back. (A) A locomotion tracking system for cyborg insects, which uses a camera to track the insect's position, transmitting the positional data to the host for recording; (B) a host that acquires and modifies the control signal parameters and obtains the locomotion trajectory data from the cyborg insect locomotion tracking system; (C) schematic representation of the locomotion control system for cyborg insects; (D) illustration of electrode implantation and stimulation points. The red arrows indicate magnified views, and the black arrows denote positional annotations.

In this study, female Madagascar hissing cockroaches (*Gromphadorhina portentosa*) were utilized, with a mean body length of 5.53 ± 0.82 cm (*N* =12) and an average weight of 6.12 ± 1.43 g (*N* =12), as illustrated in Fig. 1D. Selected for their docile nature and substantial size, these cockroaches are proven to be ideal subjects for locomotion control systems in cyborg insect technology. Literature suggests that female specimens are favored due to their superior performance in experiments involving insect motion control [39].

The subjects were maintained in well-ventilated containers and provided with a diet comprising various fruits, vegetables, and breadcrumbs to ensure optimal nutrition. In order to inhibit the proliferation of bacteria and fungi, a strict weekly cleaning schedule was established, accompanied by regular health assessments to reduce the likelihood of infection and illness. The ethical treatment of these insects is ensured throughout the entirety of the research process, with their welfare being prioritized at all times.

### Collection of electrophysiological signals from cockroach cerci

The high-precision electrophysiological equipment LabAide IX-BIO4 (World Precision Instruments) was utilized to collect electrophysiological signals from the cerci of cockroaches (Fig. S1). The cockroaches' cerci were meticulously trimmed, and electrodes made from tungsten wire were implanted into the cerci. The implantation process was carried out with care to minimize tissue damage and ensure optimal signal recording. After implantation, zinc phosphate cement (polycarboxylate zinc water cement) was applied to secure the electrodes at the connection points, ensuring a stable and enduring recording setup.

The electrophysiological signal acquisition system was activated on the host computer, and the software’s preview function was used to confirm the collection of the electrophysiological signals. This step was crucial for verifying the reliability of the connection between the biological signal acquisition device and the electrodes. Subsequently, the parameters of the electrophysiological signal acquisition equipment and processing system, including the acquisition channels and modes, were adjusted to an appropriate operational state for signal recording.

Initially, electrophysiological signals were collected while the cockroaches were resting. Subsequently, the cockroaches were induced to perform straight-line crawling behavior using food as a stimulus. The marking feature of the software was utilized to record the time points at which the cockroaches were transitioned into a moving state, with annotations being provided to describe the movement status of the cockroaches during the experiment. This approach enabled the real-time monitoring of the nervous system and muscle activity of the cockroaches during both rest and motion, providing valuable insights into the electrophysiological characteristics of the cerci.

### Design of the wireless control backpack

**Fig. 2.**
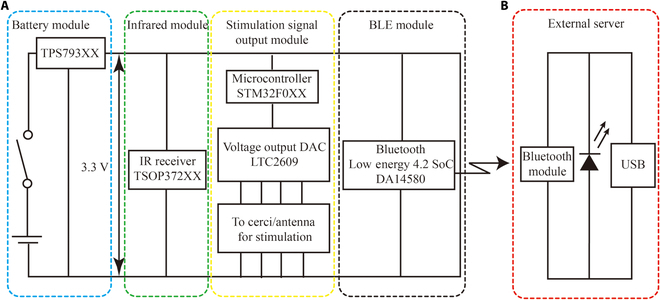
The schematic diagram of the cyborg insect locomotion control system based on electrical stimulation. (A) Schematic diagram of the signal transmission circuit; (B) schematic diagram of the signal reception circuit in the external server.

To provide electrical stimulation to the cockroach's cerci, this study designed a wireless control backpack, as shown in Fig. 2. The circuit size of the wireless control backpack is 20 mm × 35 mm and consists of 4 key components: power management, infrared remote control signal reception, control signal output, and a low-power BLE (Bluetooth Low Energy) communication module. The total weight of the backpack is 2.07 g, powered by a 3.7-V lithium-polymer battery (FLYOUNG, 3.7 V, 180 mAh, 6 g). Including the battery, a fully assembled backpack weighs 6.207 g, which is within the effective payload limit of insects and thus safe to use [15].

During the experimental process, the battery could supply approximately 2 h of the operating time. The experimental design incorporated an infrared (IR) receiver (Vishay, TSOP37238) to achieve remote control output functionality. By using a microcontroller STM32F051K8U6 (ST, 32 MHz, 64 KB of Flash) as the main control core, the backpack could provide up to 4 independently controllable stimulation channels. A 16-bit high-precision voltage-output digital-to-analog converter (DAC) chip LTC2609 (ADI, Quad 16 Bit Rail-to-Rail DACs with an Inter-Integrated Circuit [I^2^C] interface) was used to construct the high-precision output voltage module of the wireless control backpack to obtain biphasic analog electrical stimulation signals at the output. The STM32 controlled the chip LTC2609 through I^2^C communication, ultimately producing the stimulation signals.

### Procedure for backpack mounting and electrode implantation

The cockroach is placed in a container and administered CO_2_ anesthesia, which facilitates its subsequent fixation during the experimental procedure. Trimming the cockroach’s cerci, a tungsten wire electrode (Kedou Brain-Computer Technology Co., Suzhou, China, polyimide-coated, 50 μm diameter) was inserted to a depth of 10 mm (Fig. S2). Before insertion, both ends of the electrode were burned to remove the insulation coating. The reference electrode was implanted into the middle position of the cockroach’s second abdomen, at a depth of 5 mm, and firmly secured with beeswax [40]. The choice of tungsten for the electrode material is attributed to its unique physical properties [41]. Tungsten’s high melting point and excellent electrical conductivity allow it to withstand high-temperature environments while effectively conducting electrical currents during stimulation. Moreover, the corrosion resistance and chemical stability of tungsten ensure that the electrode–tissue interface does not degrade noticeably over extended periods of electrophysiological experiments, thereby ensuring the consistency and reliability of the electrical stimulation signals. The polyimide-coated tungsten wire electrode, with a diameter of 50 μm, not only provides good mechanical strength but also minimizes inflammatory responses upon tissue contact, which is crucial for improving the success rate of the experiments and the welfare of the insects.

After successful electrode implantation, the control backpack was securely attached to the cockroach’s back using double-sided tape to ensure that the wires did not cause short circuits, as shown in Fig. 1C. Finally, the power switch was activated.

It is important to note that the cockroach’s dorsal surface is covered with a layer of wax, making it difficult for most adhesives to adhere the control backpack to the cockroach. Therefore, we roughened the back surface with sandpaper to increase surface texture and promote adhesion. The control backpack was then fixed to the cockroach’s back using double-sided foam tape. Following the surgery, the cockroach was placed back into the rearing box container with the lid open to enhance air circulation, allowing the cockroach to rest for 1 h before conducting experiments.

### Design of stimulation signals

**Fig. 3.**
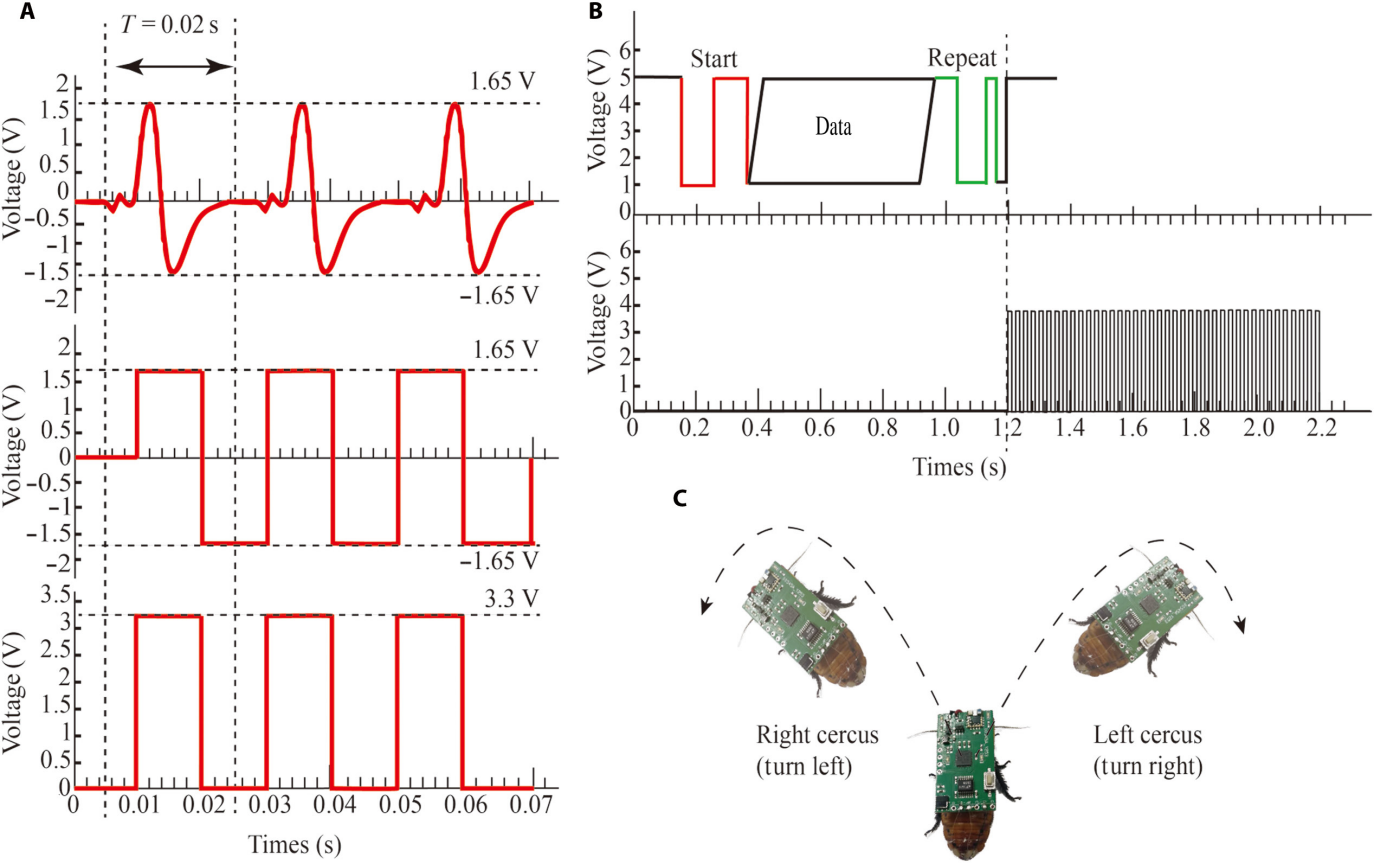
Stimulation signals and how they control cyborg insect movement. (A) Waveform of the stimulation signal controlling the turning of the cyborg insect. (B) The control backpack generates monophasic square signals based on the received remote control commands. (C) Tthe stimulation signal changes the direction of movement of the cyborg insect. Stimulating the left cercus of the insect results in a right turn, whereas stimulating the right cercus causes an insect to turn left.

Figure 3A depicts the waveforms of the 3 signal types under investigation, with the biphasic analog signal shown at the top, the biphasic square signal below it, and the monopolar square signal at the bottom. Notably, all 3 signals exhibit a peak-to-peak amplitude of 3.3 V and a consistent period of *T* = 0.02 s, corresponding to a frequency of 50 Hz. This frequency choice is based on prior research demonstrating its effectiveness in eliciting neural responses in insect models [42]. The design of the biphasic analog signal includes a shorter positive phase duty cycle to mitigate the risk of electrochemical damage at the tissue–electrode interface (Fig. S3), a common concern with prolonged direct current application. This design, coupled with the signal's ability to mimic natural action potentials of neurons, is expected to enhance its efficacy in evoking neural responses. In addition, the negative phase of the biphasic analog signal provides an electron flow path opposite to that of the positive phase, which mitigates the charge accumulation effect at the electrode–tissue interface that occurs during the positive phase, thus further reducing the possibility of tissue damage. This charge-balancing mechanism is anticipated to offer the biphasic analog signals greater reliability and consistency compared to both biphasic square wave and monopolar signals.

### Locomotion control of cockroach

To accurately evaluate the effectiveness and consistency of our cyborg insect locomotion control system, we meticulously designed and executed a series of experiments. These experiments were conducted on a custom-built platform, as illustrated in Fig. 1A. For real-time monitoring of the insects, we affixed a prominent red marker on the dorsal side of each cyborg insect. This marker considerably improved the accuracy of our locomotion tracking system, enabling precise recording of the insects’ movement trajectories during turning experiments. The trajectory data captured during these experiments were recorded in video format for subsequent analysis.

**Fig. 4.**
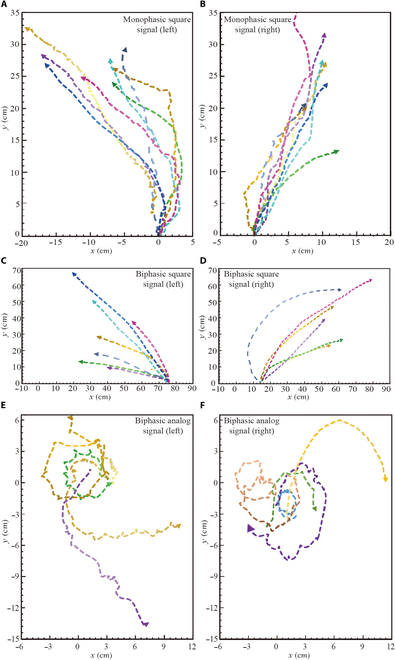
Partial locomotion trajectories of the cyborg insect. At the beginning of all experiments, the cockroaches were placed at the origin (0,0) position, with their heads facing the positive *Y*-axis. (A) Trajectory during the left turn under a 50-Hz monophasic square signal; (B) trajectory during the right turn under a 50-Hz monophasic square signal; (C) left turn trajectory under a 50-Hz biphasic square signal; (D) locomotion trajectory during the right turn under a 50-Hz biphasic square signal; (E) locomotion trajectory during the left turn under a 50-Hz biphasic analog signal; (F) locomotion trajectory during the right turn under a 50-Hz biphasic analog signal.

Using Tracker (Fig. S4), we thoroughly processed the experimental videos to extract the locomotion coordinates of the cyborg insects. We then carefully plotted the trajectories of successful turning control experiments in a coordinate system, as demonstrated in Fig. 4. In each trial, we closely monitored the responses and movement trajectories of the cockroaches when subjected to directional stimulation signals.

In order to ensure reliable results and prevent performance degradation due to fatigue, a 120-min testing period was allocated for each cockroach. During testing, a 1-s signal was applied 10 times to the right cercus of each cockroach, followed by 10 applications to its left cercus. A 5-min break was integrated between each signal to allow for recovery. Additionally, a 30-min rest was scheduled after completing the 10 trials on the right cercus before initiating the 10 trials on the left cercus. In this study, we tested 3 different types of electrical signals, each designed to elicit specific behavioral responses from the cockroaches. To verify the stability and reproducibility of these signals, we conducted 4 sample tests for each signal type, using different cockroaches for each sample, resulting in a total of 12 cockroach samples.

**Fig. 5.**
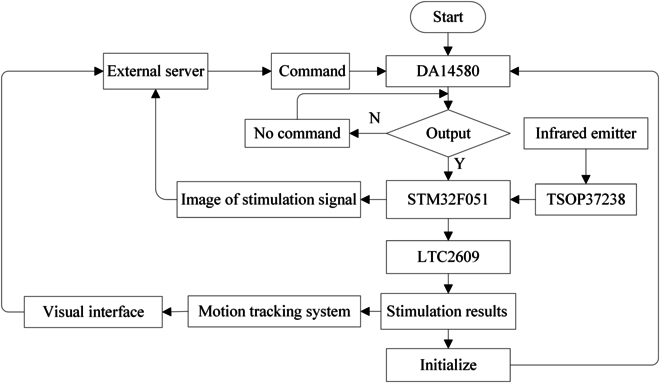
The block diagram of the wireless control systems. The system consisted of 2 components: software and hardware; the hardware included a wireless control backpack, a locomotion trajectory tracking system, and an external server; the software components were installed on the computer of the external server.

For each sample, we applied 10 electrical stimuli to both the left and right cerci to evaluate the impact of the stimulation on the cockroach's behavior. The turning experiments aimed to establish a precise mapping relationship between the electrical stimulation signals and the behavior of the cyborg insect. The output of the control signals was managed through an infrared remote controller, while the parameters of the stimulation signals were transmitted by the BLE module in the stimulation backpack, sent to an external server, and dynamically displayed on the locomotion trajectory of the cyborg insect, as illustrated in Fig. 5. When adjustments to the stimulation signal parameters were necessary, corresponding modifications were made through commands sent by the BLE module via the external server.

Initially, we employed a traditional 50-Hz monophasic square signal to stimulate the right cercus of the cyborg insects and meticulously observed and recorded their movement states. This experiment was repeated 10 times on 4 different cockroach samples. Subsequently, the left cerci of the same cockroach samples were also stimulated with the same electrical experiments. The recorded trajectories from these experiments are depicted in Fig. 4A and B.

Next, to explore the effects of changes in signal type on insect behavior, we transitioned from a monophasic square to a biphasic square signal (as shown at the bottom of Fig. 3A) and conducted the same experiments, with the corresponding trajectories illustrated in Fig. 4C and D.

Finally, we conducted turning experiments using a biphasic analog signal (as shown at the top of Fig. 3A), and the recorded trajectories during the experimental process are presented in Fig. 4E and F. Through this series of rigorous experimental steps, we were able to comprehensively analyze and understand the effects of different electrical stimulation signals on the behavior control of cyborg insects.

## Results and Discussion

### Locomotion control system of cyborg insect

The wireless control system consists of 2 primary components: a wireless control backpack and an external server, as depicted in Fig. 5. The external server mainly provides users with a graphical interface, enabling them to visualize the current waveform of the electrical stimulation signal, which records the locomotion trajectory of the cyborg insect.

The external server communicates bidirectionally with the wireless control backpack via BLE. When the external server is not transmitting electrical stimulation signals, it can utilize the Over-The-Air functionality to remotely update the code of the BLE chip within the wireless control backpack. The BLE chip communicates with the main control chip through an I^2^C interface, thereby permitting the modification of output signal parameters.

Action potentials in the neurophysiological control of insect behavior are considered to operate at the millivolt level, based on our experiments involving the collection of electrophysiological signals from the cerci of cockroaches and a review of the relevant literature [32]. This suggests that a high-precision controllable voltage output is a crucial component of the insect locomotion control system. Notably, a voltage output resolution as fine as 0.05 mV can be achieved by our system when operated at a voltage of 3.3 V.

The workflow of the wireless control system is illustrated in Fig. 5. The external server predefines the stimulation signals it intends to output through the BLE to the wireless control backpack, which then enters a standby mode. Upon receiving the output command, the wireless control backpack delegates the output of the stimulation electrical signal to the LTC2609 chip, while transmitting the waveform of the output signal to the screen of the external server. After being stimulated, the cyborg insect initiates movement, and the locomotion tracking system (refer to Fig. 1A) captures and records the insect's locomotion trajectory on the external server.

### Electrophysiological signal of cockroach cerci

**Fig. 6.**
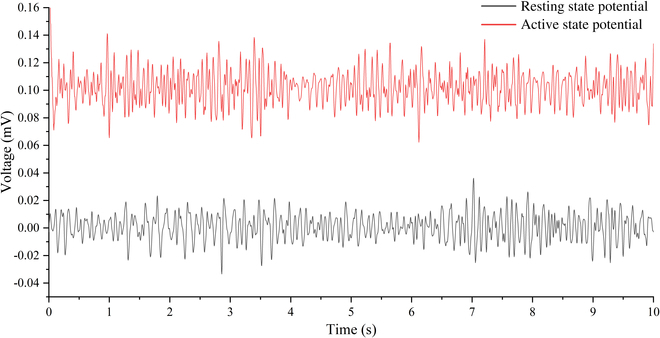
Schematic representation of electrophysiological signals from the right cercus of a cockroach. The black line represents the potential graph in a resting state, while the red line represents the potential graph in an active state.

The electrophysiological signal data collected were processed using a Fast Fourier Transform band-pass filter. The filter was set with a lower cutoff frequency of 5 Hz and an upper cutoff of 75 Hz, resulting in filtered data that were plotted in Fig. 6. This plot shows that the amplitude of the electrophysiological parameters of the cockroach’s cerci during locomotion is observed to be approximately 0.06 to 0.12 mV, while the amplitude of these parameters in a resting state is observed to range between +0.02 mV and −0.02 mV. Spectral analysis reveals that in both the resting and moving states of the cockroach, the predominant frequency distribution is concentrated around 8 Hz (Fig. S5). Upon examination of the potential graphs, it was determined that the electrophysiological signals of the cockroach's cerci exhibit a biphasic nature, characterized by a zero-voltage integral, indicative of an analog signal. This discovery provides valuable insights for the design of our stimulation signals. When developing applications for insect behavior control, it is suggested that the biphasic nature and charge balance of the signals should be taken into account to ensure optimal interaction with the natural electrophysiological properties of the target organism.

### Impact of electrical stimulation signals on steering success rate

In these experiments, the locomotion control of a cyborg insect was achieved through electrical stimulation of its sensory organs [42]. In the experiments, aside from using one channel as a common electrode, 2 channels were employed to stimulate the left and right cerci of a cockroach. This design ingeniously simulated bilateral stimulation scenarios that might occur in the natural environment, providing a comprehensive understanding of the response mechanism of the Madagascar hissing cockroach. Specifically, stimulating the left cercus triggered an avoidance response, causing the cockroach to exhibit a right-turn behavior. Similarly, stimulating the right cercus induced a left-turn behavior, as shown in Fig. 3C. In this study, a clear deviation greater than a 20°-angle deviation threshold toward the side opposite to the electrical stimulation was considered an effective steering control after the wireless backpack received the steering command. This study examined the occurrences of steering behavior induced by monophasic square signals, biphasic square signals, and biphasic analog signals separately, and the obtained results are presented in Table.

**Table.** The cockroach responses to different stimulus signals. Each signal was applied to 4 cockroaches, with 10 repetitions of left/right stimulation for each cockroach. A total of 12 cockroaches participated in the experiment.

Stimulation signal	Stimuli site	Turn left	Turn right	No response
Monophasic square stimulation	Right cercus	30	–	10
	Left cercus	1	31	8
Biphasic square stimulation	Right cercus	36	–	4
	Left cercus	–	35	5
Biphasic analog stimulation	Right cercus	39	–	1
	Left cercus	–	38	2

In the experiments involving 10 instances of electrical stimulation with a monophasic square signal on the left cercus, the cockroach’s turning response gradually weakened with the stimulation number, and in some experiments, there was even no response at all (Fig. S6). A similar phenomenon was observed in the stimulations of the right cercus with a monophasic square signal. In 80 electrical stimulation experiments conducted on 4 different cockroaches using the monophasic square signal (*n* = 80, 40 on the left cercus, and 40 on the right cercus), a correct turning response occurred in 61 experiments. When the biphasic square signal was used in the turning stimulation in 80 experiments, this phenomenon was alleviated, achieving approximately 71 correct turning responses, and in only 9 experiments, the cockroach did not exhibit the correct turning response. When the biphasic square signals were converted into biphasic analog signals with the same frequency and applied to the tail of a cockroach, a noticeable turning response occurred in each experimental sample in all 10 unilateral electrical stimulations. The statistical results of the experiments indicated success rates of 76.25%, 88.75%, and 96.25% for turning control with monophasic square signals, biphasic square signals, and biphasic analog signals, respectively.

The cockroach's cerci serve as sensory organs, reacting to external stimuli, such as air currents and object contact. The experimental results effectively demonstrate that electrical stimulation of the cerci could trigger escape responses in cockroaches. It was observed that the success rate of cockroach turning induced by monophasic square signals was 76.25%. However, with the increase in the number of experiments, instances of turning failures became more frequent, which could be attributed to the charge accumulation around the stimulation point, which would damage the tissue cells around the stimulation point, thus causing the cockroach to become less sensitive to monophasic square signals [25]. In contrast, due to the integration of voltage over time, which resulted in zero charge accumulation, the biphasic square signals exhibited a lower impact of the charge accumulation around the stimulation point, providing a higher success rate in turning control.

The experimental results indicate that the application of charge-balanced biphasic electrical stimulation signals can mitigate the damage caused by charge accumulation in cyborg insects, potentially extending the operational lifespan of these biobots. This suggests that biphasic analog signals employing a charge-balanced strategy hold considerable promise in the development of long-term, safe, and effective electrical stimulation strategies for cyborg insects. However, the optimal stimulation parameters for biphasic analog signals under varying locomotion control requirements may necessitate in-depth exploration based on specific conditions. Future research should focus on individualizing these parameters to align with specific operational demands and environmental variables, aiming to enhance the precision and reliability of the stimulation protocols. Moreover, the exact mechanisms by which the cockroach nervous system interprets and responds to biphasic electrical stimulation remain not fully understood. Further investigation into these neural processes is essential for refining the stimulation techniques. Additional research must be conducted to better understand the neural response to electrical stimuli, which will be vital for improving stimulation technology and maximizing the utility of biphasic signals in cyborg insect applications.

### Influence of electrical stimulation signals on locomotion parameters

**Fig. 7.**
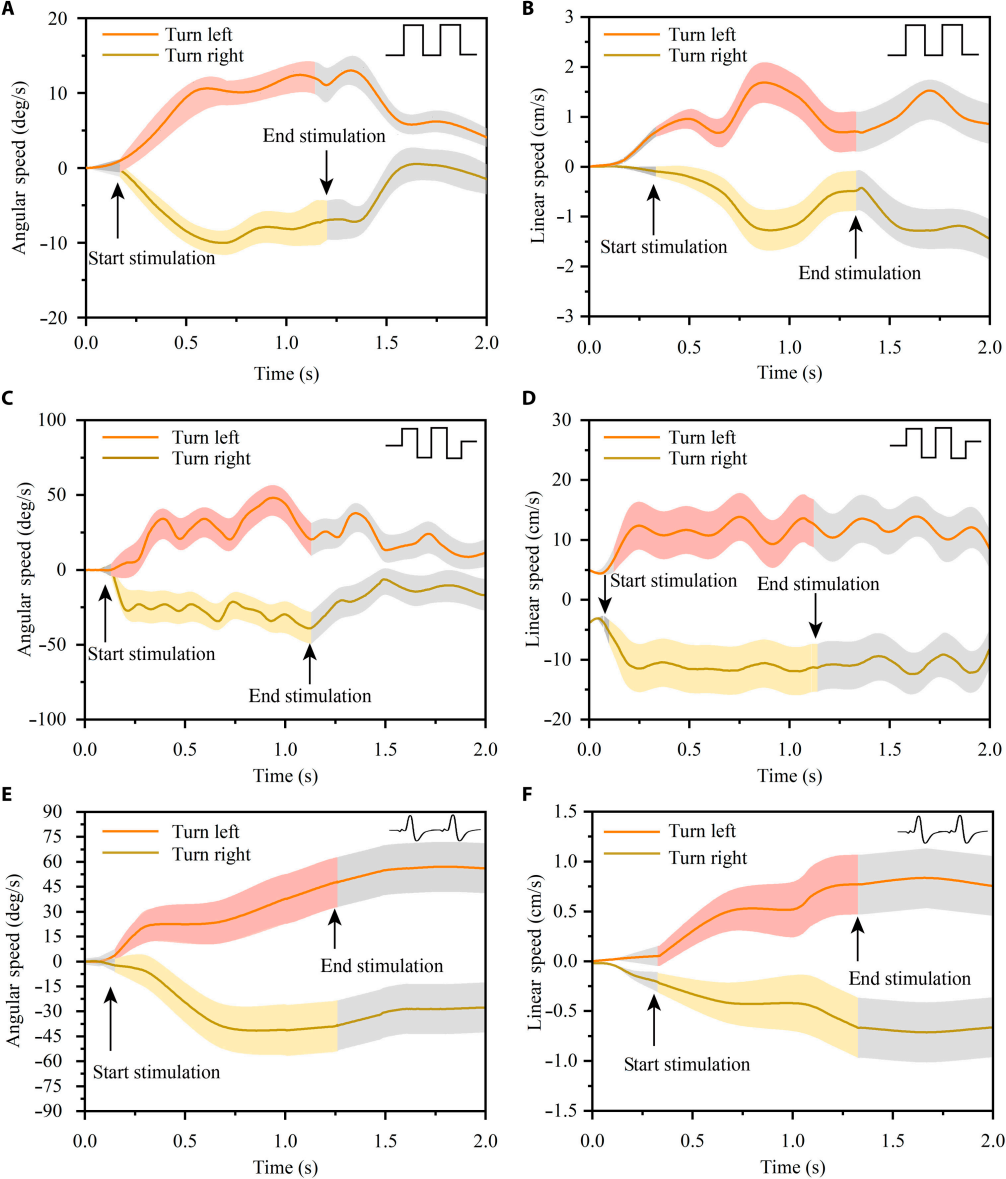
The locomotion data of the cyborg insect. The red line represents the electrical stimulation to the right tail whisker, inducing the leftward locomotion in the cyborg insect; the green line refers to the electrical stimulation to the left tail whisker, causing the rightward locomotion in the cyborg insect; the shaded area indicates the standard deviation, and the light-colored region indicates that no electrical stimulation signal was applied. (A) The cockroach turning angular velocity induced by a monophasic square signal. (B) The cockroach turning linear velocity induced by a monophasic square signal. (C) The cockroach turning angular velocity induced by a biphasic square signal. (D) The cockroach turning linear velocity induced by a biphasic square signal. (E) The cockroach turning angular velocity induced by a biphasic analog signal. (F) The cockroach turning linear velocity induced by a biphasic analog signal.

The angular velocity and linear velocity values during the locomotion of the cyborg insect were calculated by analyzing the trajectory maps and timelines. A set of experimental data was selected to demonstrate the turning locomotion control performance, and the relationship between the angular velocity and the linear velocity over time is shown in Fig. 7.

Using the 3 different electrical stimulation signals in the experiment, a consistent pattern in the cockroach behavior was observed. After receiving the electrical stimulation signal, both angular and linear velocities increased rapidly in 0.5 s, eventually stabilizing within a certain range. After 0.5 s from the stimulation end, the cyborg insect returned to the free-movement status, and the angular velocity decreased to zero, as shown in Fig. 7A. This indicated that the turning locomotion of the cockroach occurred immediately after the electrical stimulation and ceased upon the termination of the stimulation. Although these electrical stimuli all triggered cockroach turning movements, the specific locomotion parameters varied.

For the single-polarity square signal, after receiving the stimulation signal, the angular velocity of the cockroach increased from zero to the maximum value of 10°/s in 0.5 s, as shown in Fig. 7A; meanwhile, the linear velocity increased from zero to the maximum value of approximately 2 cm/s, as shown in Fig. 7B. After the stimulation process ended, the angular velocity returned to zero, and the linear velocity remained around 1 cm/s until the cockroach stopped moving.

When the cockroach received the biphasic square signal, the angular velocity increased from zero to the maximum value of 50°/s in 0.5 s, as shown in Fig. 7C; meanwhile, the linear velocity increased from 5 cm/s to 15 cm/s, as shown in Fig. 7D. After the stimulation, the angular velocity returned to zero, while the linear velocity remained constant.

In the case of the biphasic analog signal stimulation, the angular velocity of the cockroach increased from 15°/s to 60°/s, as presented in Fig. 7E, and the linear velocity increased from zero to 1 cm/s, as shown in Fig. 7F. After the stimulation, both angular and linear velocities remained unchanged. The comparison of locomotion data of turning induced by different signals demonstrated that the angular velocity of turning induced by the biphasic square signal was more than 5 times that induced by the monophasic square signal. Similarly, the linear velocity induced by the biphasic square signal was approximately 10 times that induced by the monophasic square signal, as presented in Fig. 6B and D. However, the turning angular velocity induced by the biphasic analog signal was consistent with that induced by the biphasic square signal, while the linear velocity was consistent with that induced by the monophasic square signal.

Therefore, compared to the monophasic signals, the biphasic signals could better induce the turning locomotion of cockroaches. Also, compared to the biphasic square signal, the analog signal induced lower linear velocity during the cockroach-turning process. This could be attributed to the milder stimulation of the cockroach cerci by the analog signal, resulting in minimal damage. Thus, for the same experimental time, the cockroaches could endure more repeated analog signal stimulation, and the repeatability of the analog signal was higher than that of the square signal.

**Fig. 8.**
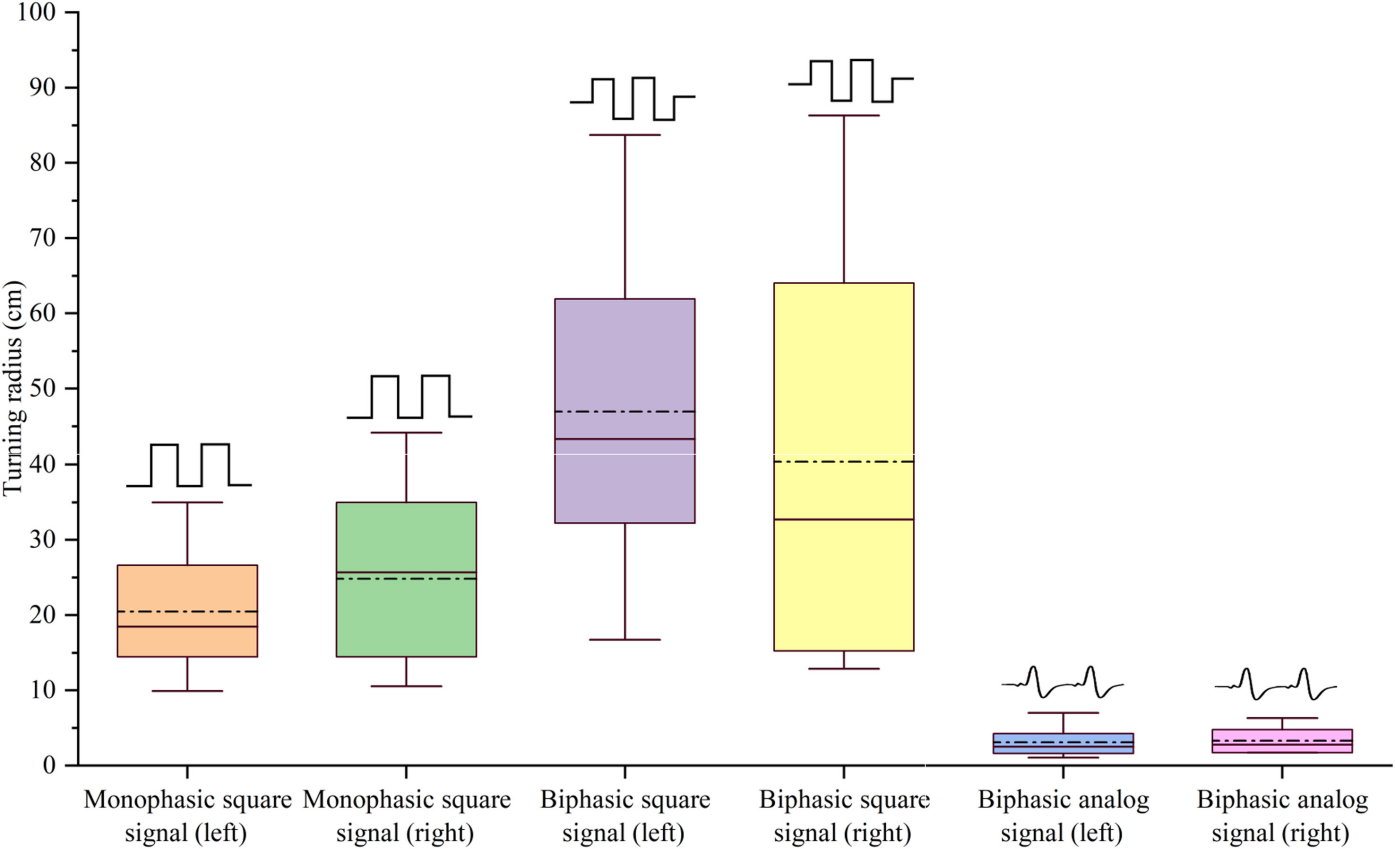
The boxplots of turning radius with different control signals. The boundaries of the box represent the 25th and 75th percentiles, and the line inside the box indicates the average value; the solid horizontal line represents the median.

All data from the experiments where the direction was successfully controlled were selected, including monophasic square signals, biphasic square signals controlling the left and right turns of cockroaches, and biphasic analog signals controlling the left turn of cockroaches. The turning radius of the cyborg insect was calculated in this study by fitting the initial curvature of the path with a circle of radius *R*, as shown in Fig. 8, where it can be seen that the average turning radius values for the left and right turns induced by the monophasic square stimulation signals were 20.5 cm and 24.7 cm, respectively. The turning radius induced by the biphasic square signals was the largest, having the average left- and right-turn radius of 46.9 cm and 40 cm, respectively. The results demonstrated that the turning radius range induced by the biphasic squares was relatively large, whereas that induced by biphasic analog signals was the smallest among all analyzed signals. Therefore, this study calculated the standard deviation of the turning radius for left and right turns induced by the biphasic square signals and biphasic analog signals. The standard deviation values of the left and right turns induced by biphasic square signals were 21.9 and 26.7, respectively, and those of left and right turns induced by the biphasic analog signals were 1.9 and 2.6, respectively. We conducted a one-way analysis of variance on the turning radius data, and the experiment indicated that there is a significant difference in the overall mean at the 0.05 level, leading to the reliable conclusion that the data are reliable (Tables S1 to S3). Thus, it could be concluded that the biphasic square signals could induce turning movements with larger radii than the monophasic square signals, but their turning radii were not stable enough to control the specific turning radii of the cyborg insects precisely. In contrast, the turning radius variations induced by the biphasic analog signals were small, resulting in a more stable turning radius value. However, the biphasic analog signals could not induce large-radius turning movements.

The experimental results indicated that in the turning stimulation experiments, the biphasic square signals with a peak value of 3.3 V, a frequency of 50 Hz, and a duty cycle of 50% exhibited superior turning control performance compared to the monophasic square signals with the same parameters. Although the turning control success rates for both experimental groups exceeded 76.25%, there was a significant change in the radius of the cockroach’s turning locomotion during each experiment. Compared to the 2 signals, the biphasic analog signals provided higher reliability. The results of multiple short-term repeated experiments showed that the cyborg insect responded consistently to each biphasic analog signal stimulus. This suggested that biphasic analog signals could induce a relatively small change in the turning radius under short-duration repetitive stimulations, while the biphasic square signals might have the potential to induce a larger turning radius.

### Limitations and future work

Despite valuable insights being yielded by our research, certain limitations exist that necessitate further elucidation. A major limitation is that despite using biphasic analog stimulation signals, the habituation of cockroaches has not been completely alleviated, which may lead to a decrease in their responsiveness to electrical stimulation over time. The sustained effectiveness of cyborg insects in prolonged operations is considerably challenged by this phenomenon. Furthermore, the potential benefits of integrating feedback mechanisms or implementing graded control systems within our cyborg insect control framework are particularly noteworthy. Such innovations could facilitate more nuanced interactions with these insects, thereby enhancing our ability to guide their movements and reactions. The advantages of current stimulation over voltage stimulation are also noteworthy. Current stimulation offers direct control over the quantity of charge delivered to the tissue, allowing precise modulation of stimulation intensity and subsequent biological responses. This approach ensures consistent charge transfer regardless of tissue impedance variations, unlike voltage stimulation, which may yield less predictable outcomes in response to such changes (Fig. S7). Additionally, the use of voltage stimulation could pose risks, such as the potential for electrolytic reactions at the electrode–tissue interface. The efficiency and long-term stability of cyborg insects could potentially be compromised by electrode corrosion or tissue damage resulting from these reactions. Conversely, current stimulation, particularly when applied with lower currents and biphasic pulses, can mitigate the risk of such electrochemical damage, thereby maintaining the long-term efficiency of charge transfer. It is crucial to acknowledge that our study did not comprehensively investigate the impact of various stimulus parameters—such as duty cycle, voltage amplitude, and electrical stimulation frequency—on cockroach locomotion control. A thorough examination of these parameters is imperative for refining control strategies for cyborg insects.

In subsequent research, we aim to address these limitations by conducting an in-depth investigation into the application of current stimulation. We will explore the integration of feedback systems to achieve more nuanced control over cyborg insect responses and conduct systematic studies to understand the impact of different stimulus parameters on insect locomotion. Furthermore, we will investigate methods to counteract habituation, potentially through adaptive stimulation protocols or by incorporating additional sensory modalities. Through these efforts, we aspire to make marked progress toward the development of more effective and dependable cyborg insect systems.

## Conclusion

This study proposes a cyborg insect locomotion control system capable of generating high-precision analog signals. The proposed system is used to investigate a reliable turning locomotion control strategy for the Madagascar hissing cockroach. The experimental results reveal that stimulating the cockroach’s cerci with a monophasic square signal at a frequency of 50 Hz, a voltage of 3.3 V, and a duty cycle of 50% can achieve a 76.25% success rate in inducing turning. When a biphasic square signal with the same parameters is used, the turning success rate increases to 88.75%. Further, it is shown that the modification of the biphasic square signal into the biphasic analog signal results in a remarkable 96.25% success rate in turning locomotion control. These results exceed the previously reported success rate of 70% for locomotion control, indicating that the charge-balanced biphasic electrical stimulation can help to protect cyborg insect tissues from cumulative damage caused by repeated monophasic stimulation, thus enhancing the reliability of cyborg insects. Moreover, in the context of cyborg insect locomotion control, biphasic analog stimulation signals have greater research importance and development potential compared to monophasic electrical stimulation signals. Therefore, in scenarios requiring high-precision locomotion control, such as cyborg insect autonomous navigation systems, it may be beneficial to consider using appropriate biphasic analog stimulation signals instead of traditional monophasic square signals to achieve better outcomes in locomotion control.

Future work could expand the functionality of the proposed insect wireless control system, modifying it into a high-quality platform for cyborg insect research. In addition, wireless control backpacks tailored for different host insects could be designed based on the proposed wireless control system.

## Data Availability

The authors confirm that the data supporting the findings of this study are available within the article or its supplementary materials.

## Supplementary Materials

Supplementary 1 Collection of electrophysiological signals from cockroach cerci Design of stimulation signals Collection and analysis of cockroach locomotion data Phenomenon of habituation to electrical stimulation in cockroaches One-way analysis of variance Profile of the induced currents Figs. S1 to S7 Tables S1 to S3 Movie S1 Reference [43]
