# Supplementary material for: Locomotion Control of Cyborg Insects by Charge-Balanced Biphasic Electrical Stimulation
Source: Cyborg Bionic Syst. 2024 Jul 5;5:0134. doi: 10.34133/cbsystems.0134 (PMC11223913; doi:10.34133/cbsystems.0134)
Supplement: Supplementary 1 — Collection of electrophysiological signals from cockroach cerci Design of stimulation signals Collection and analysis of cockroach locomotion data Phenomenon of habituation to electrical stimulation in cockroaches One-way analysis of variance Profile of the induced currents Figs. S1 to S7 Tables S1 to S3 Movie S1 Reference [43] [file cbsystems.0134.f1.zip › Supplementary Material.docx]

**Supplementary Materials**

**Collection of electrophysiological signals from cockroach cerci**

In the study, the LabAide IX-BIO4, a single-channel electrophysiological signal acquisition device tailored for small animals, was utilized (Fig. S1A). Efficient and stable data transfer was provided by this device through a direct USB connection to a computer. The integrated analog-to-digital converter within the LabAide IX-BIO4 is capable of operating at a sampling rate of up to 20 kHz, which enables the precise capture of subtle electrophysiological signals. The precision achieved at this level is essential for analyzing variations in electrophysiological signals in Madagascar hissing cockroaches across their diverse locomotive behaviors.

The LabAide IX-BIO4 was configured during the experimental procedure for the acquisition mode within the PC's host application. The neuro recording electrodes accompanying the LabAide IX-BIO4 instrument are crafted from tungsten (Fig. S1C), featuring a diameter of 50 µm. The fine size and flexibility of these electrodes facilitate their easy implantation into the biological tissues of the Madagascar hissing cockroach, while minimizing the associated tissue damage. A protective coating insulates the electrodes, with the exception of the tip, ensuring that the signal acquisition is targeted and specific.


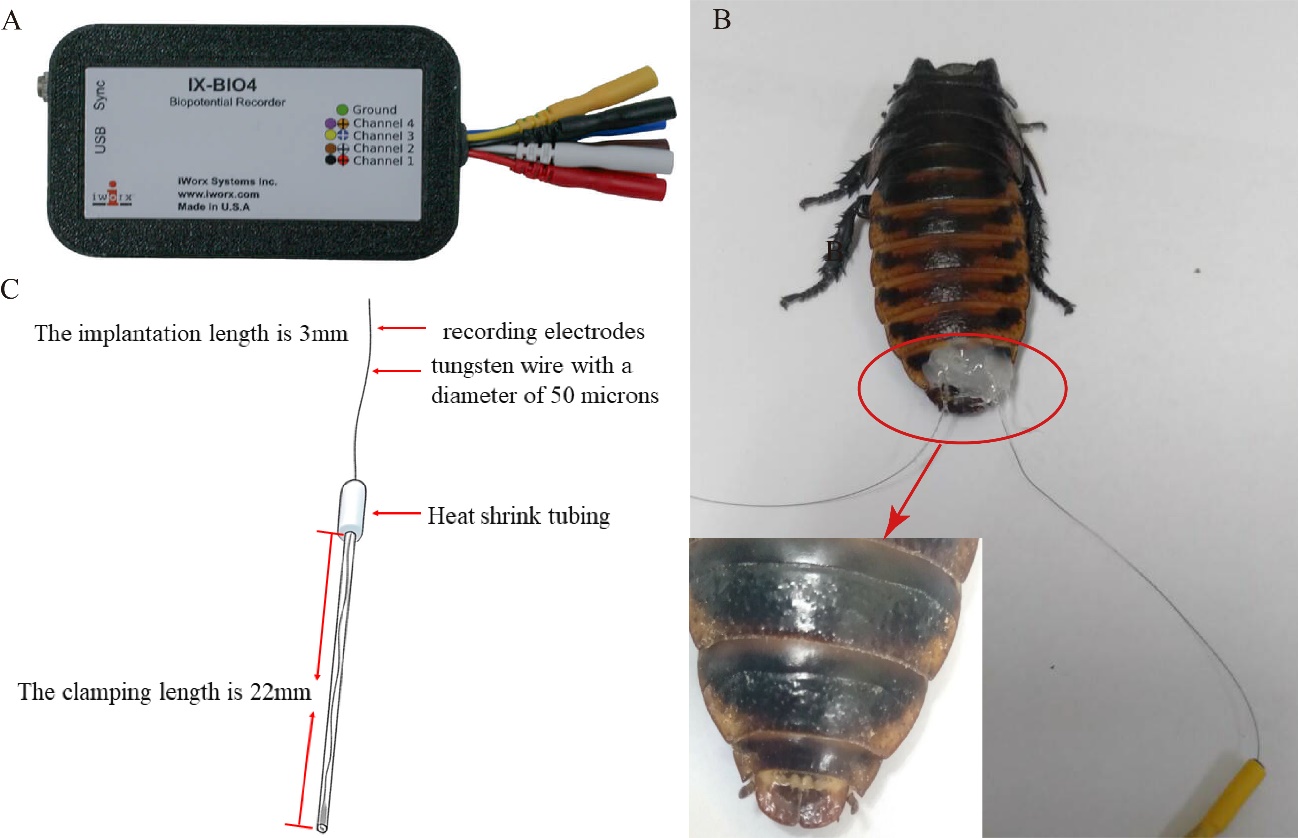


**Fig. S1. Electrophysiology Data Collection Experiment Schematic.** (A) LabAide IX-BIO4 Electrophysiology Signal Acquisition Device. (B) Cockroach cerci implanted with neural recording electrodes. Reference electrode connected to ground. (C) Tungsten Wire Neural Recording Electrode

**
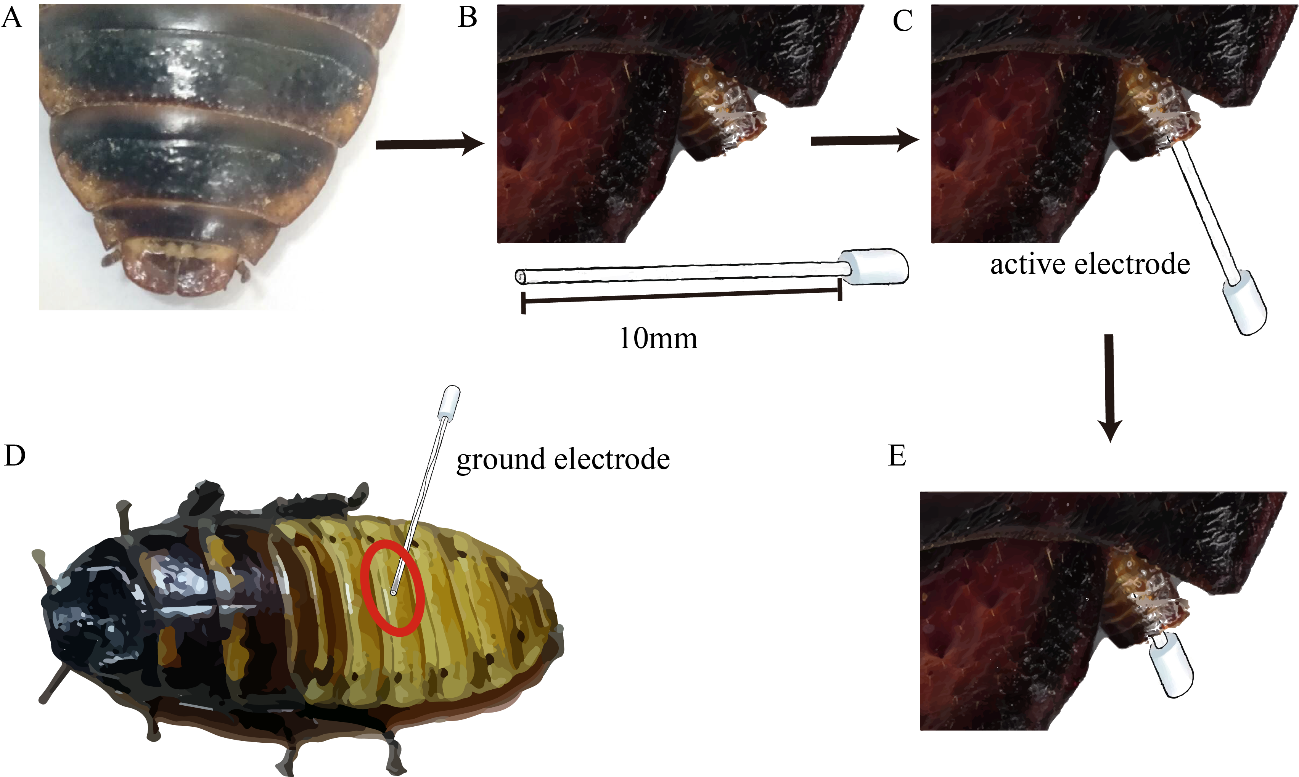
**

**Fig. S2. Electrode implantation procedure for collecting electrophysiological signals from cockroach cerci.** (A) The active electrode was implanted at the left and right cerci of the cockroach. (B) The implantation depth of the electrode at the cerci was 10 mm. (C) After trimming the cockroach's cerci, the electrode was inserted into the hollow channel of the cerci to a depth of 10 mm. (D) The reference electrode was implanted at the midpoint of the cockroach's third abdominal segment, with a depth of 5 mm. (E) After implanting the active electrode into the cerci, it was secured with zinc phosphate cement (polycarboxylate zinc water cement).

**Design of stimulation signals**

The bipolar analog stimulation signal was designed with reference to the natural characteristics of neuronal action potentials, with the expectation that these signals would effectively simulate and elicit neural responses in insects. Action potential images were obtained from publicly available scientific literature, and Engauge Digitizer software was utilized to extract data points from these images. These data points were subsequently redefined to align with our system's voltage range, specifically targeting a peak-to-peak value of 3.3 V. Through appropriate scaling and conversion of the extracted data points, a series of voltage values were generated to mimic action potentials. These values were then encoded into hexadecimal format and stored in an array for subsequent output by the digital-to-analog converter.

**
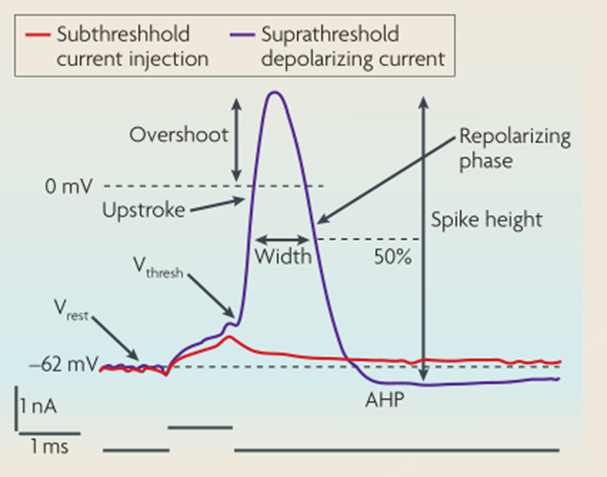
**

**Fig. S3. Anatomy of an action potential**^[44]^**.** This figure illustrates an action potential from a pyramidal neuron in the CA1 region of a rat hippocampus, triggered by a brief suprathreshold depolarizing current injection, shown in purple. A subthreshold current injection response is depicted in red. The resting membrane potential (Vrest) typically ranges from -85 mV to -60 mV, with the voltage threshold (Vthresh) at approximately -53 mV indicating the initiation point for an all-or-none firing response. The upstroke, or depolarizing phase, peaks near 0 mV, with 'overshoot' denoting the peak voltage relative to 0 mV. The spike height is measured from the peak to the resting potential or the most negative voltage during the afterhyperpolarization (AHP). The spike width is measured at half the maximal amplitude.

**Collection and analysis of cockroach locomotion data**

Tracker is a tool for video analysis and motion tracking that calculates parameters such as linear velocity and angular velocity for objects moving in a two-dimensional plane. Here is the general method by which Tracker software computes these parameters:

**1. Linear Velocity:** Linear velocity refers to the speed at which an object moves along a straight path. In Tracker, linear velocity is typically calculated by measuring the displacement of an object between consecutive frames. For two consecutive points in time, *t_1_​* and *t_2​_*, linear velocity *v* can be computed using the following formula:

$\text{v}=\frac{D}{\Delta t}=\frac{x_{2}-x_{1}}{t_{2}-t_{1}}$ (1)

Where *D* is the total distance the object moves over the time interval *Δt*, and *x_1_*_​_ and *x_2_*_​_ are the positions of the object at times *t_1_*_​_ and *t_2_*​, respectively.

2. **Angular Velocity**: Angular velocity refers to the rate at which an object rotates around a certain point. On a two-dimensional plane, if an object rotates about its center, Tracker can calculate angular velocity by measuring changes in the orientation of the object's bounding box or feature points. For two consecutive points in time, angular velocity ω can be computed using the following formula:

$\omega=\frac{\Delta\theta}{\Delta\text{t}}$ (2)

Where *Δθ* is the change in angle of the object over the time interval.

The tracker analyzes image sequences from videos to automatically identify and track the location and motion of selected objects. The software provides built-in algorithms to calculate various motion parameters and allows users to select and configure the objects of interest. The steps to use Tracker are as follows:

1. Tracking Setup: First, you need to configure. the tracking parameters in the Tracker, select an appropriate tracking model (such as point tracking or polygon tracking), and define the object of interest.

2. Video Playback: Play the video and manually or automatically select the tracking object in each frame. The Tracker will use the chosen tracking algorithm to track the object's motion in the video sequence

3. Data Recording: The Tracker will automatically record the position data of the tracking object. This data typically includes the x and y coordinates at each time point.


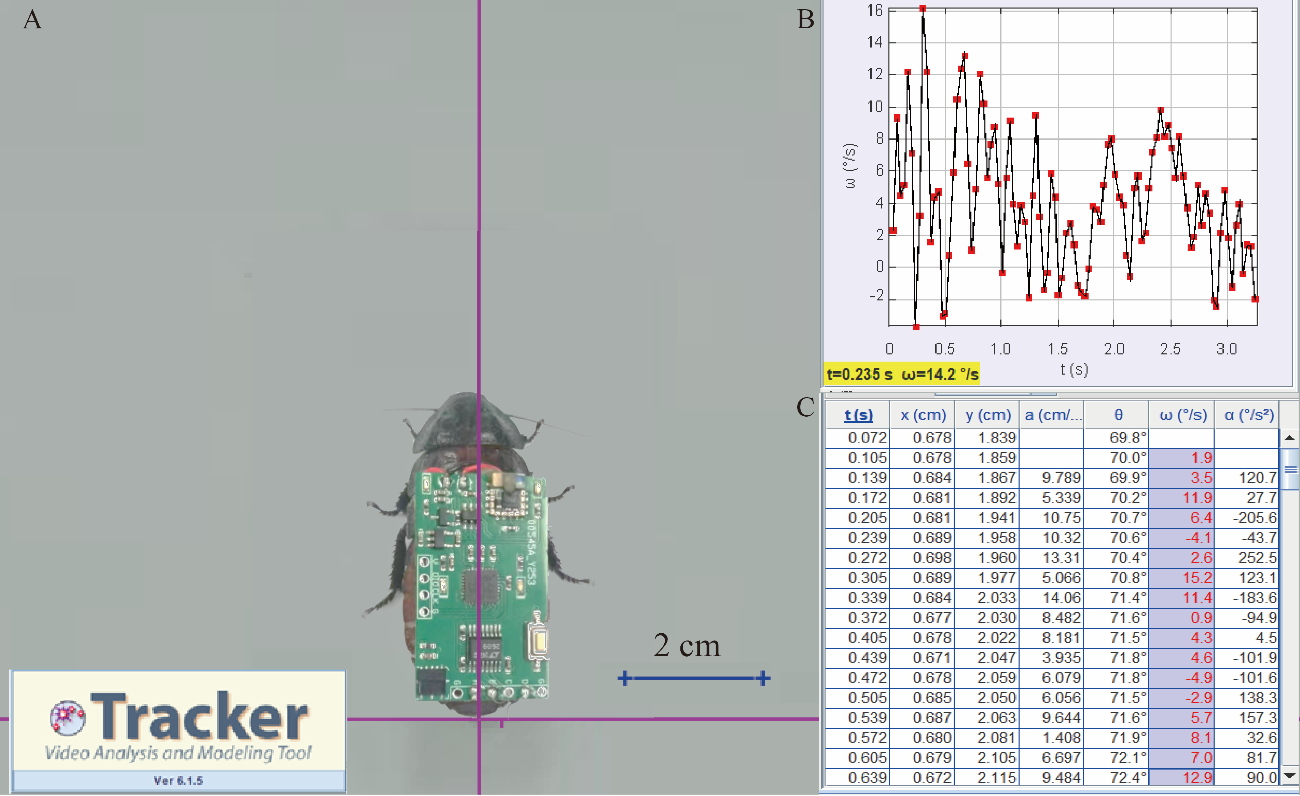


**Fig. S4. Schematic Representation of Tracker.** (A) Video Playback Panel: Displays the imported video, allowing users to scrub through frames and visualize the tracking process in real time. (B) Data Visualization Window: Allows users to customize and graphically represent the data for drawing motion trajectories. (C) Data Output: Presents real-time tracking data, including the tracked object's position coordinates, velocity, and acceleration.

**Spectral analysis of electrophysiological signals**

In this study, meticulous collection of electrophysiological signals was performed utilizing the high-precision LabAide IX-BIO4 bioelectricity acquisition device. Preprocessing and subsequent spectral analysis of the signals were conducted with the aid of MATLAB. For the spectral analysis, implementation of the Hann window function was executed to ensure optimal frequency resolution, thereby guaranteeing the precision of the outcomes. The analysis revealed that the primary frequency distribution for the cockroaches was consistently centered at approximately 8 Hz, a pattern observed regardless of the cockroaches' state, whether at rest or in motion. As the frequency increased, there was a general decline in the power spectral density within the low-frequency band. Notable differences in the spectral characteristics were not observed between the stationary and active states of the cockroaches..


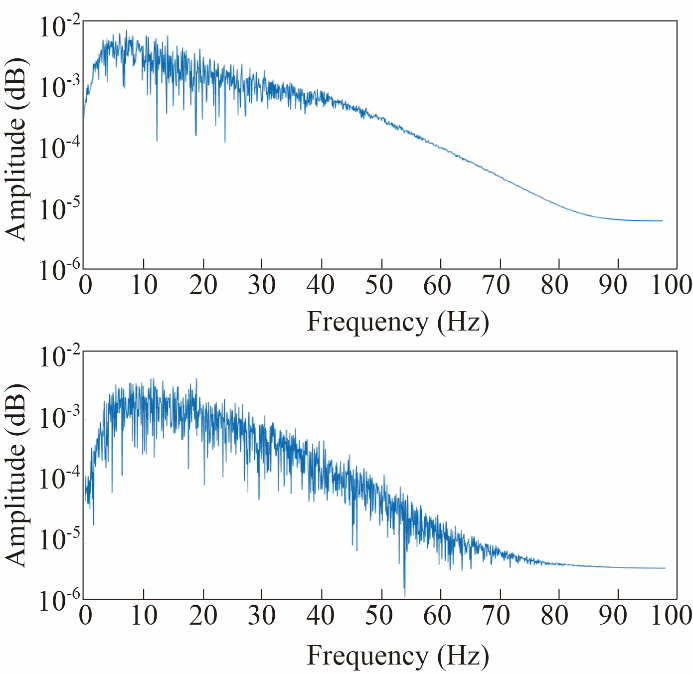


**Fig. S5. Spectral Characteristic Analysis of Electrophysiological Signals.** This figure illustrates the spectral characteristic analysis of electrophysiological signals recorded from the right cercus of cockroaches during resting and active states. The top panel shows the spectral plot during the resting state, while the bottom panel displays the spectral plot during the active state.

**Phenomenon of habituation to electrical stimulation in cockroaches**

Upon receipt of the steering command by the wireless backpack, a deviation of more than 20 degrees towards the side opposite to the electrical stimulation is deemed to represent effective steering control. In experiments where a monopolar square signal was applied for 10 instances of electrical stimulation to the left cercus, the turning response of the cockroach was observed to gradually weaken with an increase in the number of stimuli.


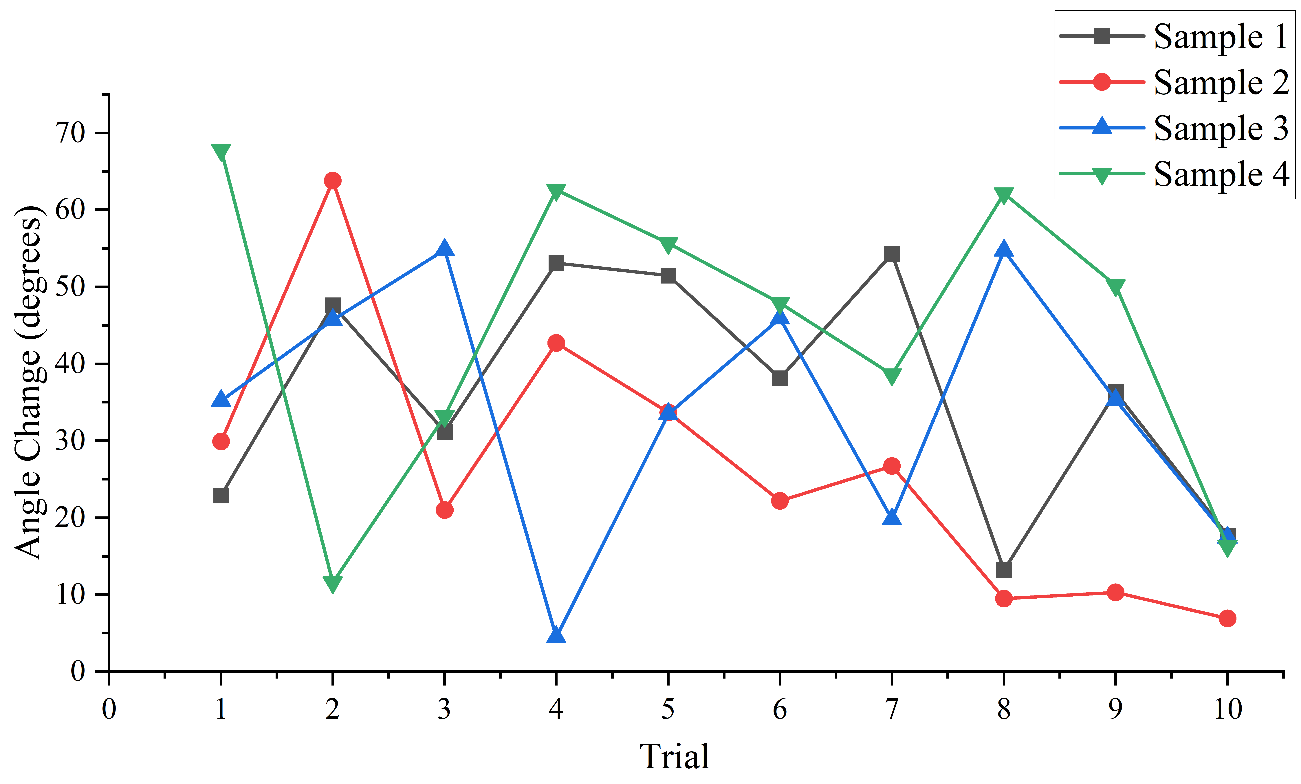


**Fig. S6. Angular Changes in Response to Electrical Stimulation Across Trials in Cockroach Samples.** The figure shows the angular changes of four individual cockroach samples in response to electrical stimulation during 10 consecutive trials. Each sample was subjected to a unipolar square signal on the left cercus, and the angle change was measured in degrees. A gradual decrease in the angular response is observed for all samples, suggesting a potential habituation effect.

**One-way analysis of variance**

To evaluate the impact of stimulus signals with varying parameters on the control of cockroach turning motion, a one-way analysis of variance (ANOVA) was conducted. Initially, it was confirmed that all data adhered to the normal distribution. Subsequently, the data were categorized into two distinct groups, designated as 'left turn' and 'right turn'. A one-way ANOVA was then executed on each of these groups.

**Table S1. Normality Test**

|  | DF | Statistic | p-value | Decision at |
| --- | --- | --- | --- | --- |
| Monophasic square signal(left) | 8 | 0.93 | 0.54 | Can't reject normality |
| Monophasic square signal(right) | 7 | 0.95 | 0.74 | Can't reject normality |
| Biphasic square signal(left) | 8 | 0.96 | 0.81 | Can't reject normality |
| Biphasic square signal(right) | 8 | 0.83 | 0.06 | Can't reject normality |
| Biphasic analog signal(left) | 8 | 0.90 | 0.27 | Can't reject normality |
| Biphasic analog signal(right) | 8 | 0.84 | 0.08 | Can't reject normality |

**Table S2. Descriptive Statistics**

|  | N Analysis | N Missing | Mean | Standard Deviation | SE of Mean |
| --- | --- | --- | --- | --- | --- |
| Monophasic square signal(left) | 8 | 0 | 0.54 | 8.63 | 3.05 |
| Monophasic square signal(right) | 7 | 1 | 0.74 | 21.90 | 7.74 |
| Biphasic square signal(left) | 8 | 0 | 0.81 | 1.96 | 0.70 |
| Biphasic square signal(right) | 8 | 0 | 0.06 | 12.02 | 4.54 |
| Biphasic analog signal(left) | 8 | 0 | 0.27 | 29.69 | 10.50 |
| Biphasic analog signal(right) | 8 | 0 | 0.08 | 2.58 | 0.91 |

**Table S3. Overall ANOVA. At the 0.05 level, the population means are significantly different.**

|  | DF | Sum of Squares | Mean Square | F Value | Prob＞F |
| --- | --- | --- | --- | --- | --- |
| Mode | 5 | 13233.20 | 2646.64 | 9.87 | 3.00E-6 |
| Error | 41 | 10988.69 | 268.02 |  |  |
| Total | 46 | 24221.89 |  |  |  |

**Profile of the induced currents**

In the realm of cyborg insect research, voltage stimulation and current stimulation are recognized as prevalent methodologies for the manipulation of insect movement. However, it has been observed that voltage stimulation does not consistently translate into the anticipated current levels, as depicted in Fig. S7, a discrepancy potentially attributable to interference at the electrode-tissue interface. In response to this observation, an assessment of the induced current emanating from voltage stimulation signals was undertaken, revealing a misalignment with the applied voltage stimulation parameters. Given these insights, a proposition is put forth that the utilization of current stimulation may provide a more precise control strategy. This approach directly regulates the electrical charge flow through biological tissues, thereby facilitating a heightened level of consistency and predictability in the domain of insect movement control.


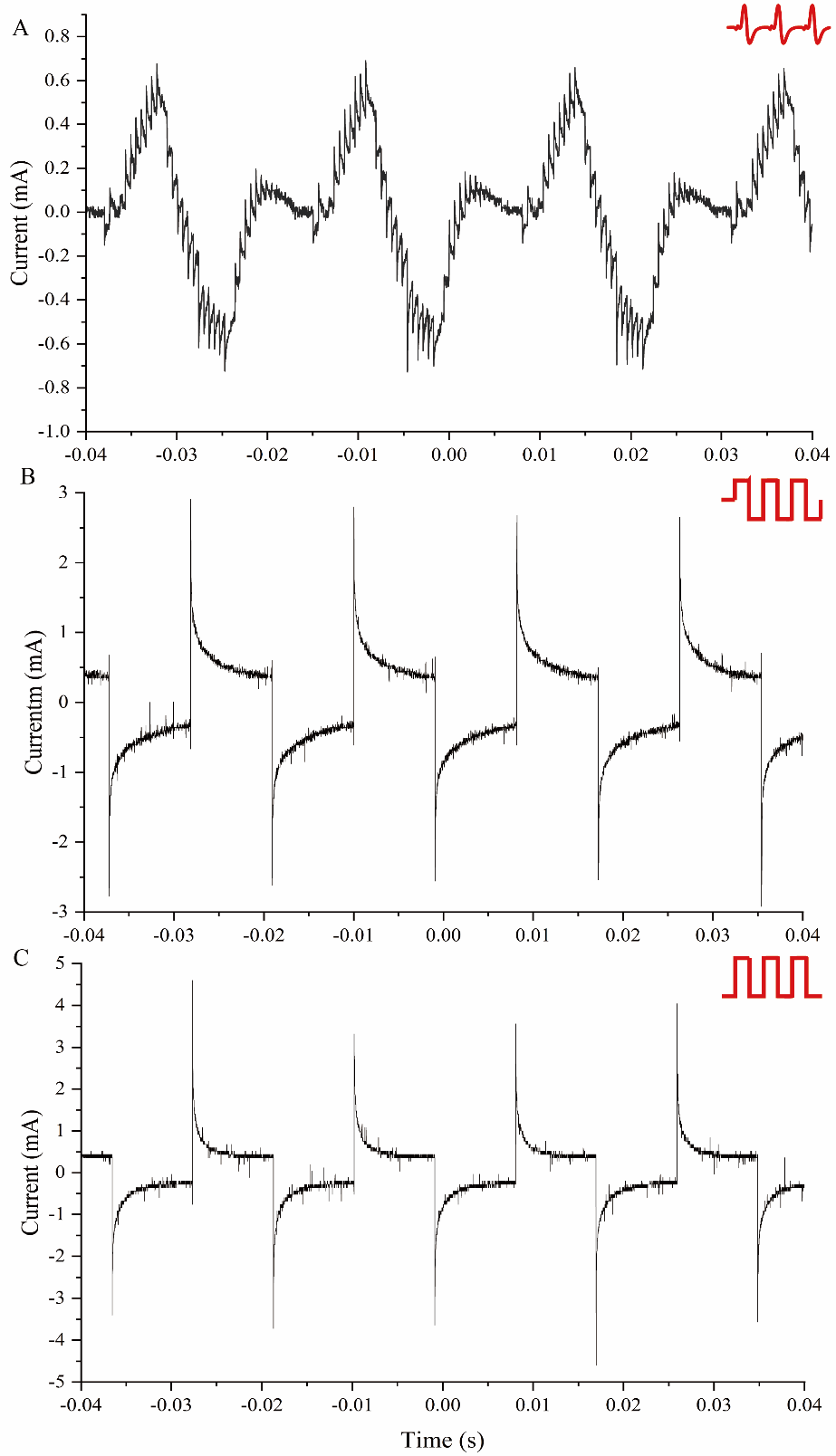


**Fig. S7. The induced current of different electrical stimulation signals.**  (A) Induced current of a biphasic analog signal. (B) Induced current of a biphasic square signal. (C) Induced current of a monopolar square signal.

Figure S1 to S7#

Tables S1 to S#

Movies S1#
